# Supplementary material for: Identification of glutathione transferase (GST P1) inhibitors via a high-throughput screening assay and implications as alternative treatment options for breast cancers
Source: PLoS One. 2025 Jul 24;20(7):e0319904. doi: 10.1371/journal.pone.0319904 (PMC12289066; doi:10.1371/journal.pone.0319904)
Supplement: S1 File — (DOCX) [file pone.0319904.s001.docx]

**Supplementary material**

**Identification of glutathione transferase (GST P1) inhibitors via a high-throughput screening assay and implications as alternative treatment options for breast cancers.**

Sarah A. P. Pereira^1,2^, Jonathan Vesin^3^, Marc Chambon^3^, Gerardo Turcatti^3^, M. Lúcia M. F. S. Saraiva^1^ and Paul J. Dyson^2^*

*^1^ LAQV, REQUIMTE, Departamento de Ciências Químicas, Faculdade de Farmácia, Universidade do Porto, Rua Jorge Viterbo Ferreira, nº 228, 4050-313 Porto, Portugal.*

*^2^ Institut des Sciences et Ingénierie Chimiques, École Polytechnique Fédérale de Lausanne (EPFL), 1015 Lausanne, Switzerland.*

*^3^ Biomolecular Screening Facility, École Polytechnique Federale de Lausanne (EPFL), 1015 Lausanne, Switzerland.*

***Figure S1 –*** *Optimization of the percentage of DMSO and ethanol used in the reaction.*


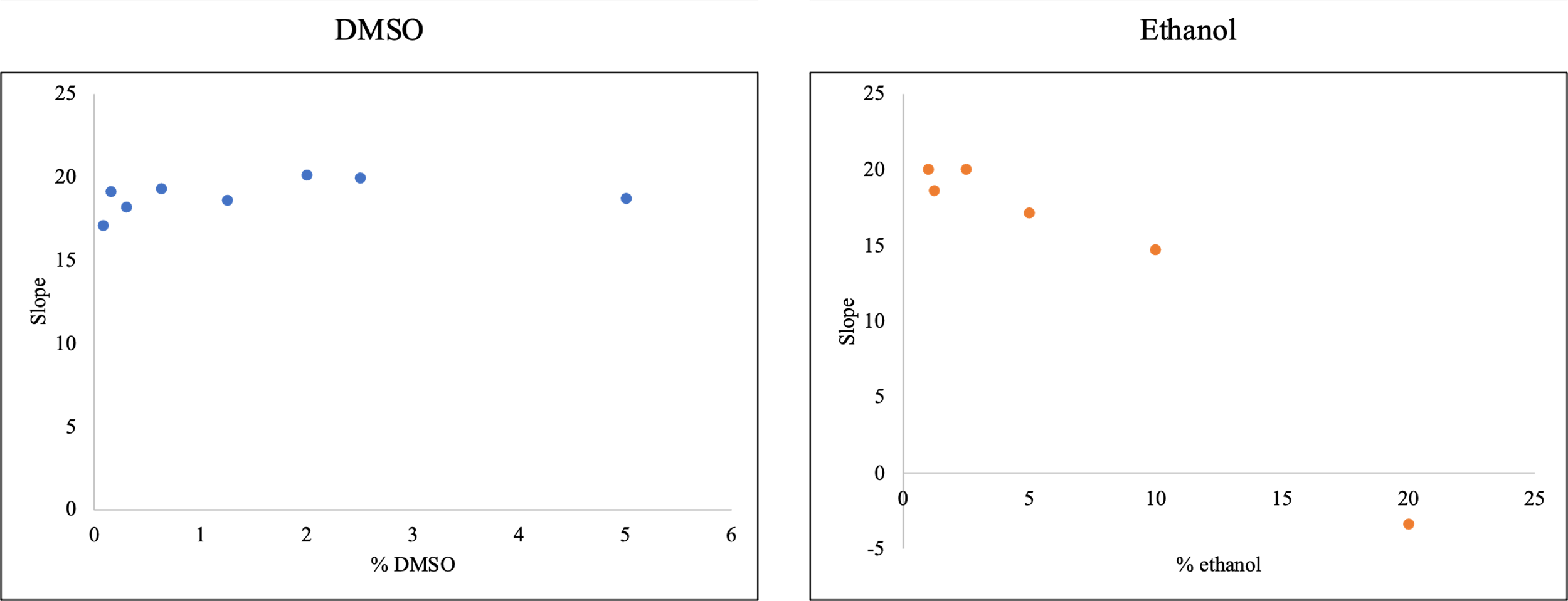


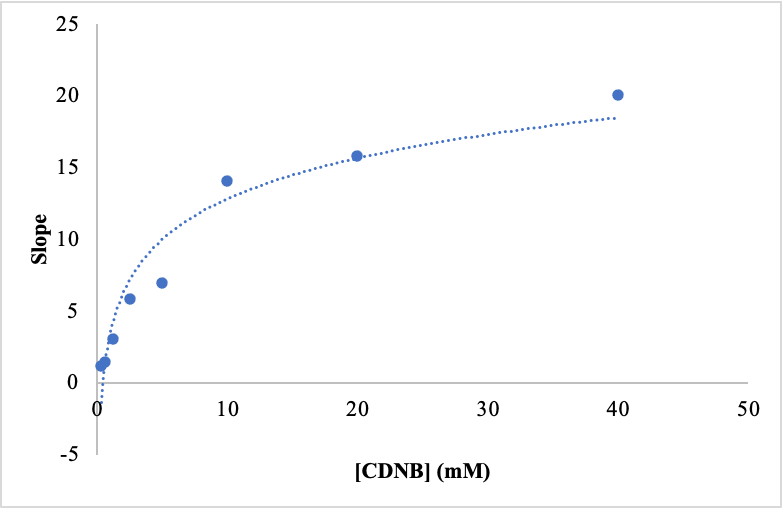
***Figure S2 -*** *Calibration curve of the GST P1 enzymatic reaction.*

***Figure S3 –*** *384-well plate assay design strategy.*


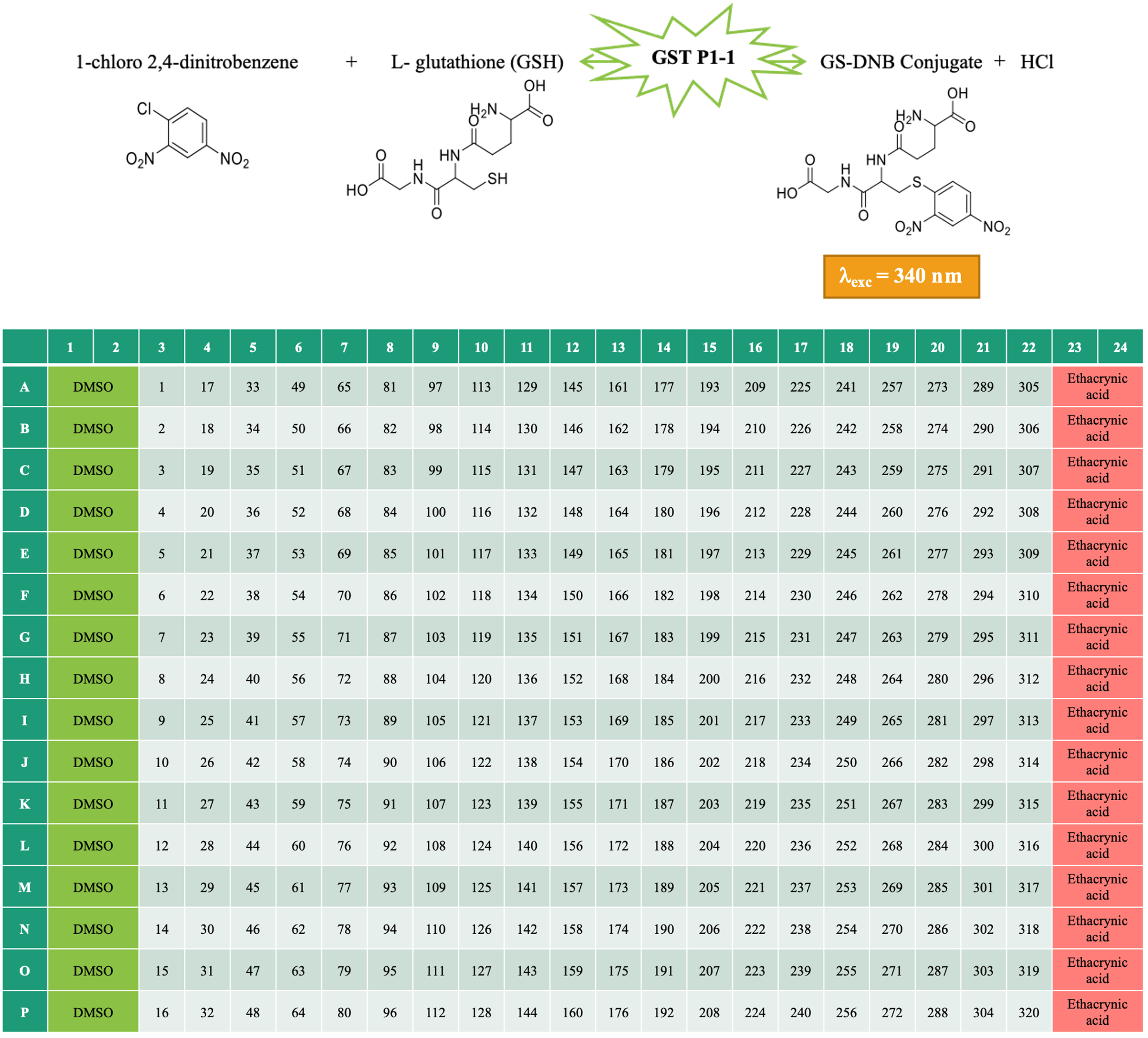


***Table S1 –*** *First 80 hit compounds obtained in the 384-well plate assay according to the score.*

| **Compound** | **Score** | **ScoreSD** |
| --- | --- | --- |
| TCID | 1.094 | 0.016 |
| Hexachlorophene | 1.077 | 0.035 |
| Cryptotanshinone | 1.037 | 0.032 |
| GW3965 (hydrochloride) | 1.027 | 0.054 |
| N-demethylantipyrine | 1.019 | 0.032 |
| Bithionol | 1.007 | 0.041 |
| Rse-bengal | 0.996 | 0.073 |
| Merbromin | 0.993 | 0.027 |
| AS-2034178 | 0.982 | 0.011 |
| Tanshinone IIA sulfonate (sodium) | 0.981 | 0.014 |
| CD-1530 | 0.979 | 0.053 |
| PSB-06126 | 0.962 | 0.021 |
| Edaravone | 0.958 | 0.071 |
| Nastorazepide | 0.958 | 0.065 |
| ZM323881 (hydrochloride) | 0.946 | 0.002 |
| CD437 | 0.939 | 0.012 |
| Rose-bengal-lactone | 0.937 | 0.062 |
| Embelin | 0.935 | 0.022 |
| MK-5108 | 0.933 | 0.058 |
| ER-50891 | 0.927 | 0.017 |
| Protoporphyrin IX | 0.914 | 0.015 |
| CHF5074 | 0.911 | 0.086 |
| nTZDpa | 0.909 | 0.031 |
| AS-252424 | 0.907 | 0.050 |
| IOWH-032 | 0.901 | 0.087 |
| PRT4165 | 0.890 | 0.019 |
| IPA-3 | 0.881 | 0.009 |
| SDZ-220-040 | 0.871 | 0.049 |
| Sal003 | 0.865 | 0.028 |
| Chicago sky blue 6B | 0.863 | 0.004 |
| Lasalocid | 0.863 | 0.039 |
| PI3K inhibitor X | 0.863 | 0.011 |
| PD-118057 | 0.858 | 0.073 |
| GW627368 | 0.854 | 0.134 |
| Ethacrynic acid | 0.850 | 0.002 |
| MNS | 0.850 | 0.009 |
| EN460 | 0.837 | 0.038 |
| Tiplaxtinin | 0.836 | 0.040 |
| Avasimibe | 0.835 | 0.062 |
| Erythrosine | 0.828 | 0.032 |
| Sulfinpyrazone | 0.823 | 0.011 |
| Docosahexaenoic Acid | 0.821 | 0.082 |
| JAK3-inhibitor-V | 0.817 | 0.053 |
| TCS PIM-1 1 | 0.813 | 0.030 |
| TPPS4 | 0.809 | 0.004 |
| RTA-408 | 0.807 | 0.046 |
| AZD9496 | 0.806 | 0.169 |
| Macelignan | 0.803 | 0.077 |
| Bromosporine | 0.801 | 0.016 |
| Zafirlukast | 0.801 | 0.204 |
| Cis-9,trans-11-Conjugated-linoleic-acid | 0.796 | 0.074 |
| ZM39923 (hydrochloride) | 0.788 | 0.059 |
| Brilliant-green | 0.783 | 0.043 |
| TUG-891 | 0.783 | 0.016 |
| Evans Blue | 0.783 | 0.031 |
| AMG 837 (calcium hydrate) | 0.783 | 0.028 |
| Hypericin | 0.774 | 0.008 |
| Bonaphthone | 0.770 | 0.020 |
| Y16 | 0.765 | 0.033 |
| Palovarotene | 0.763 | 0.084 |
| Hematoporphyrin | 0.758 | 0.006 |
| NS-3623 | 0.752 | 0.005 |
| Diphencyprone | 0.744 | 0.024 |
| AT13148 | 0.738 | 0.090 |
| SR-11302 | 0.733 | 0.069 |
| 10058-F4 | 0.731 | 0.007 |
| 4EGI-1 | 0.730 | 0.012 |
| TC-I-2000 | 0.729 | 0.040 |
| Salirasib | 0.717 | 0.037 |
| BADGE | 0.715 | 0.017 |
| Ki16425 | 0.710 | 0.103 |
| GW 4064 | 0.702 | 0.196 |
| Triclocarban | 0.702 | 0.068 |
| Temoporfin | 0.699 | 0.036 |
| RQ-00203078 | 0.697 | 0.100 |
| PHT-427 | 0.696 | 0.028 |
| Anthralin | 0.694 | 0.030 |
| SKI II | 0.694 | 0.046 |
| Buclizine | 0.694 | 0.010 |

**Figure S4 –** Dose-response curves obtained for the reconfirmation assay of the 24 selected compounds.


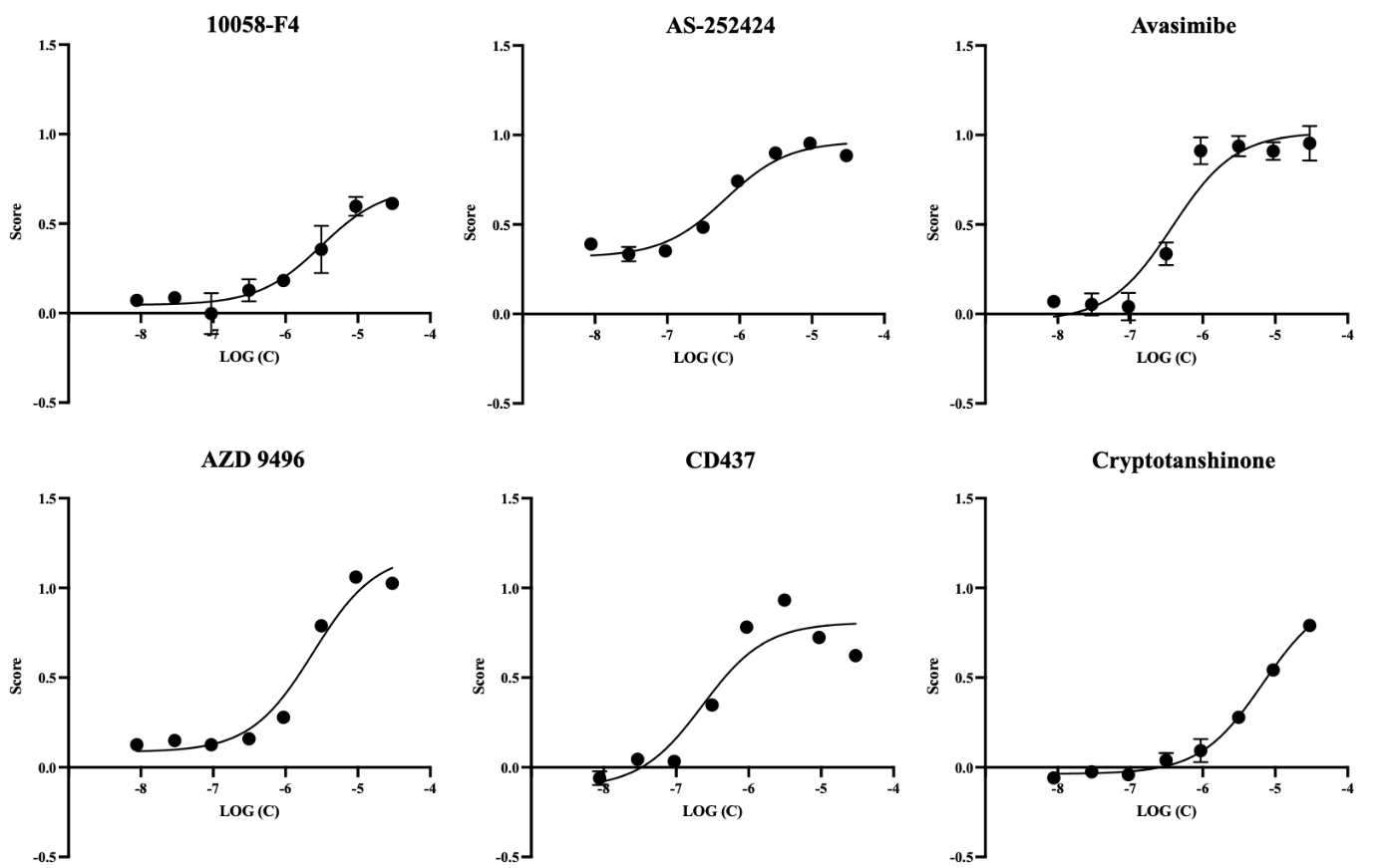

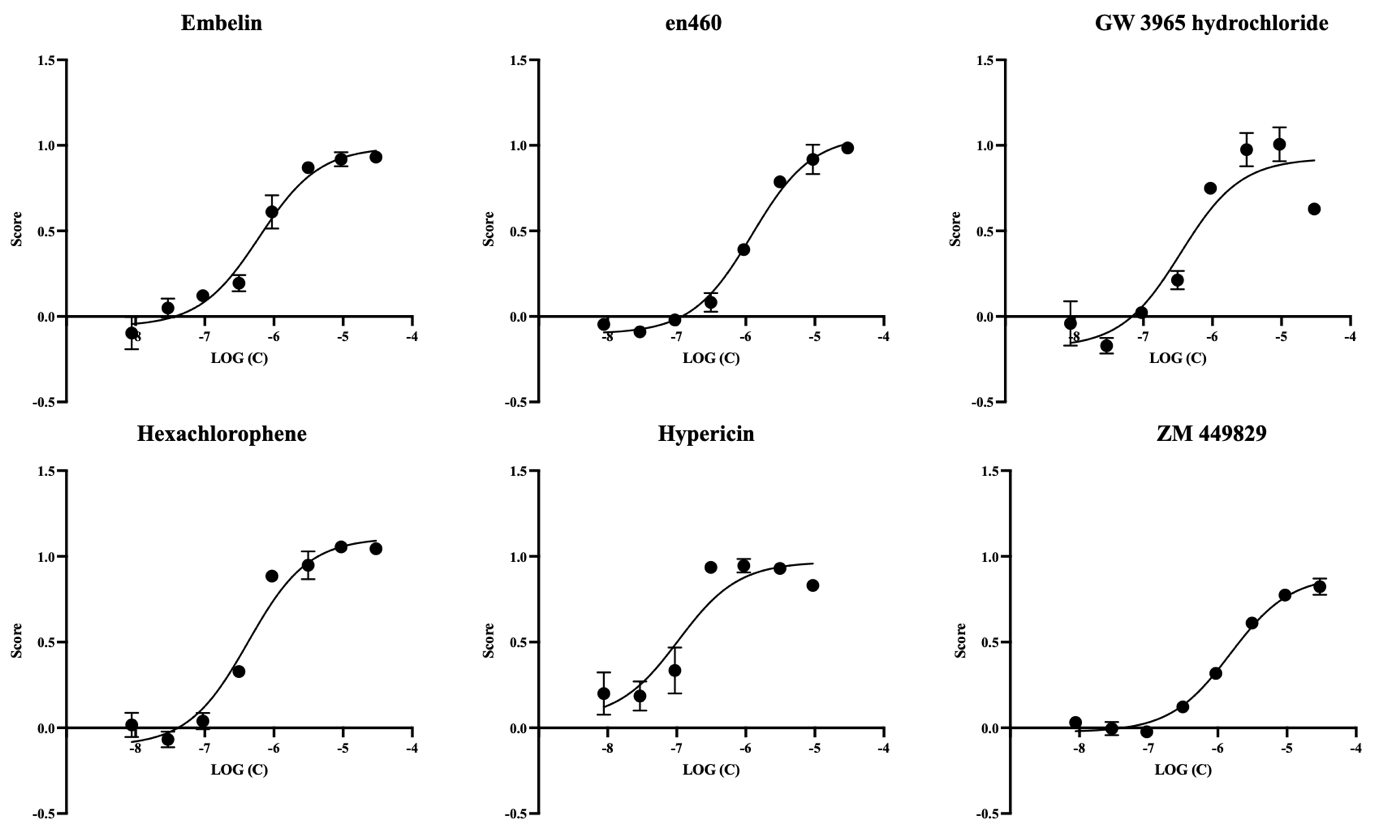

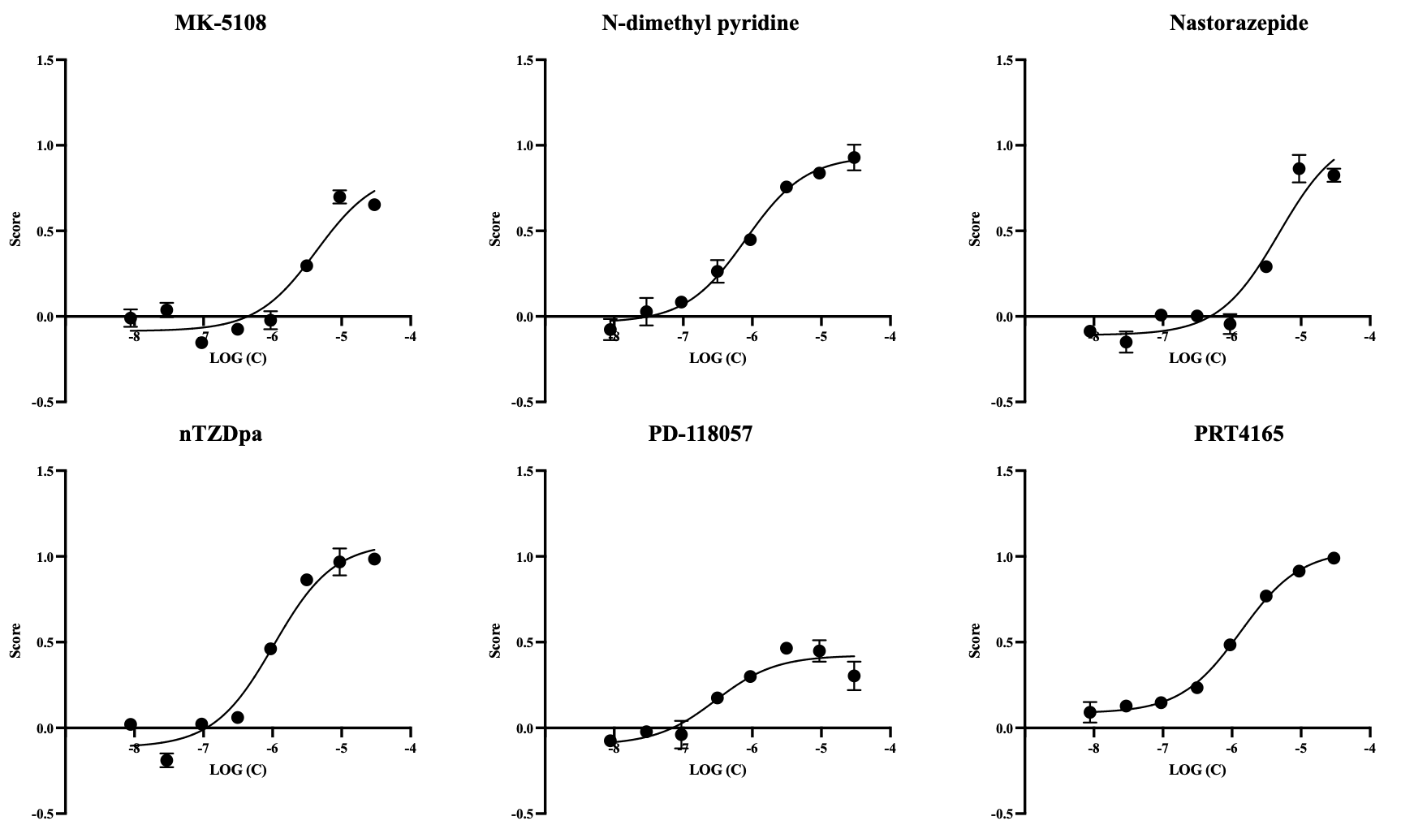

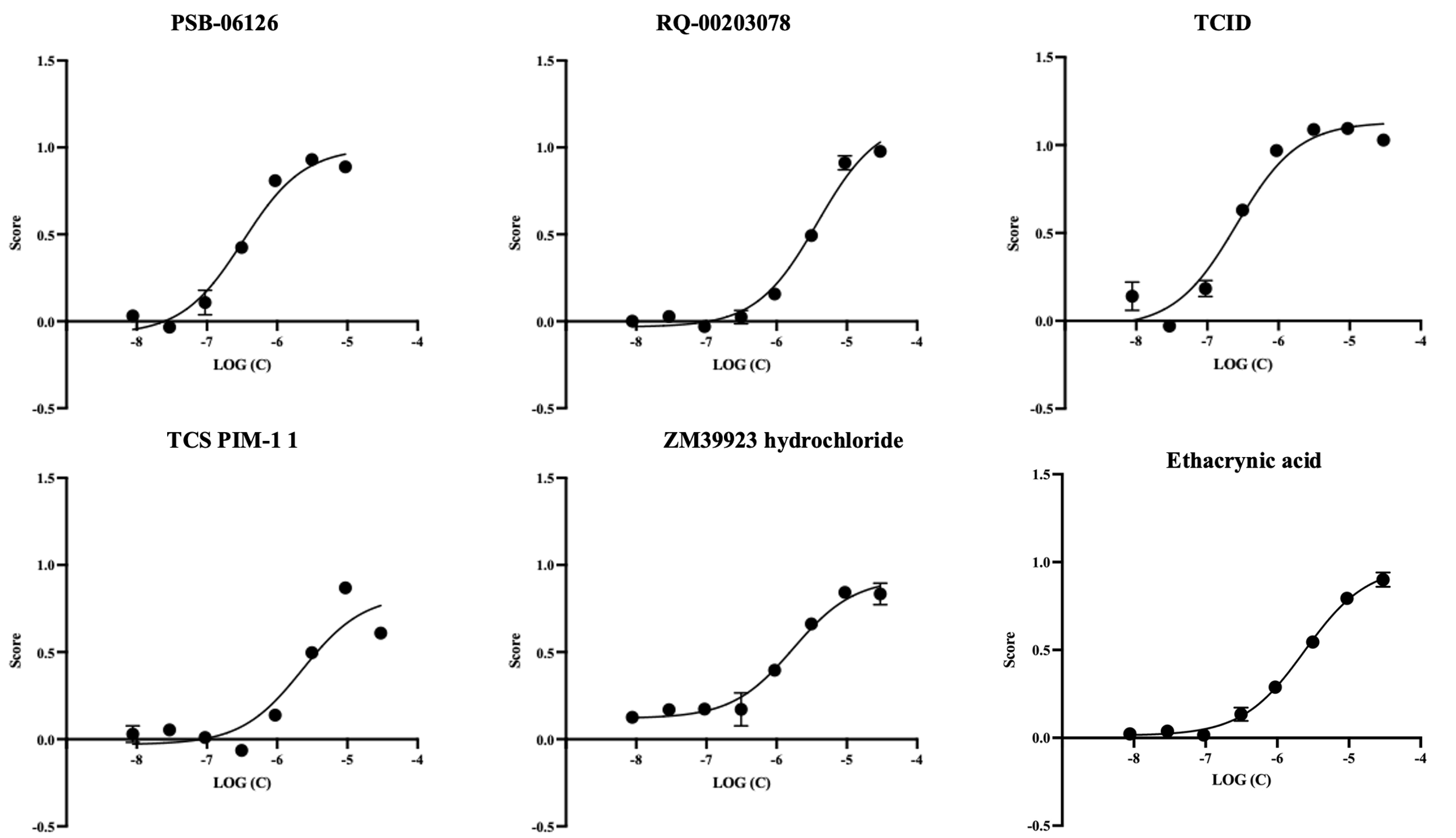


**Table S2 –** Hill slope and IC_50_ values obtained in the reconfirmation assay for the 24 compounds selected.

| **Compound** | **Hill Slope (dose-response curve)** | **IC_50_ (μM) GSTP1** |
| --- | --- | --- |
| Ethacrynic acid | 1.01 | 2.4 |
| 10058-F4 | 1.37 | 2.75 |
| Cryptotanshinone | 0.85 | 8.9 |
| ZM39923 (hydrochloride) | 1.56 | 1.5 |
| PRT4165 | 1.2 | 1.3 |
| ZM449829 | 1.3 | 1.4 |
| CD437 | 11.6 | 0.32 |
| AS252424 | 1.95 | 0.61 |
| AZD9496 | 2.23 | 2.05 |
| GW3965 (hydrochloride) | 2.48 | 0.43 |
| nTZDpa | 1.7 | 0.95 |
| RQ-00203078 | 1.6 | 3.1 |
| Hypericin | 11.6 | 0.1 |
| Embelin | 1.46 | 0.64 |
| en460 | 1.4 | 1.1 |
| Hexachlorophene | 2.2 | 0.42 |
| Avasimibe | 4.2 | 0.37 |
| MK-5108 | 13.6 | 3.2 |
| TCS PIM-1 1 | 2.5 | 2.15 |
| PSB 06126 | 3.4 | 0.29 |
| PD-118057 | 1.8 | 0.33 |
| Nastorazepide | 10.6 | 3.3 |
| 5-Methyl-2-phenyl-1,2-dihydropyrazol-3-one, 98% | 0.8 | 0.83 |
| TCID | 1.16 | 0.9 |

**Figure S5 – Western blot unprocessed raw images.**

**
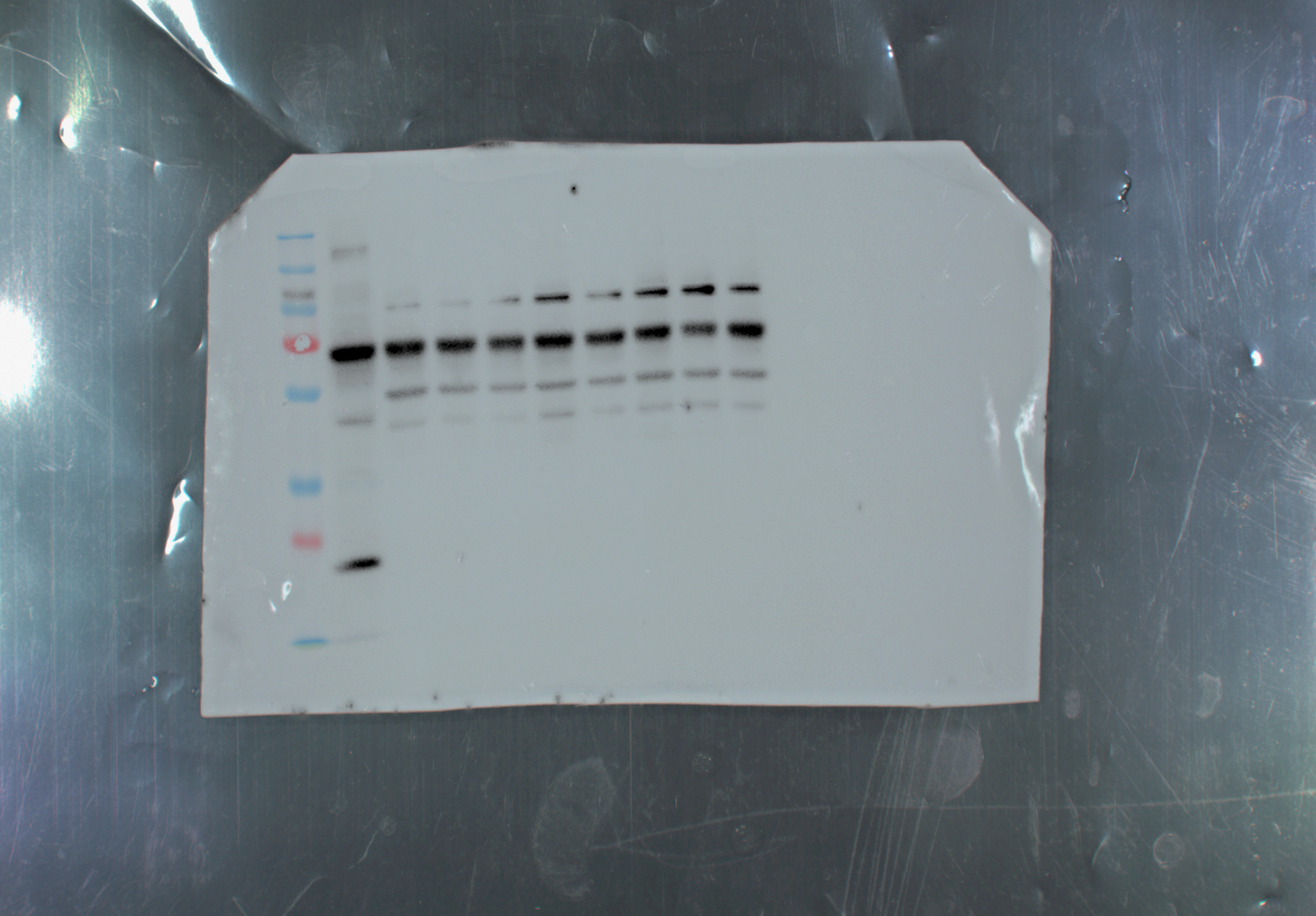
**


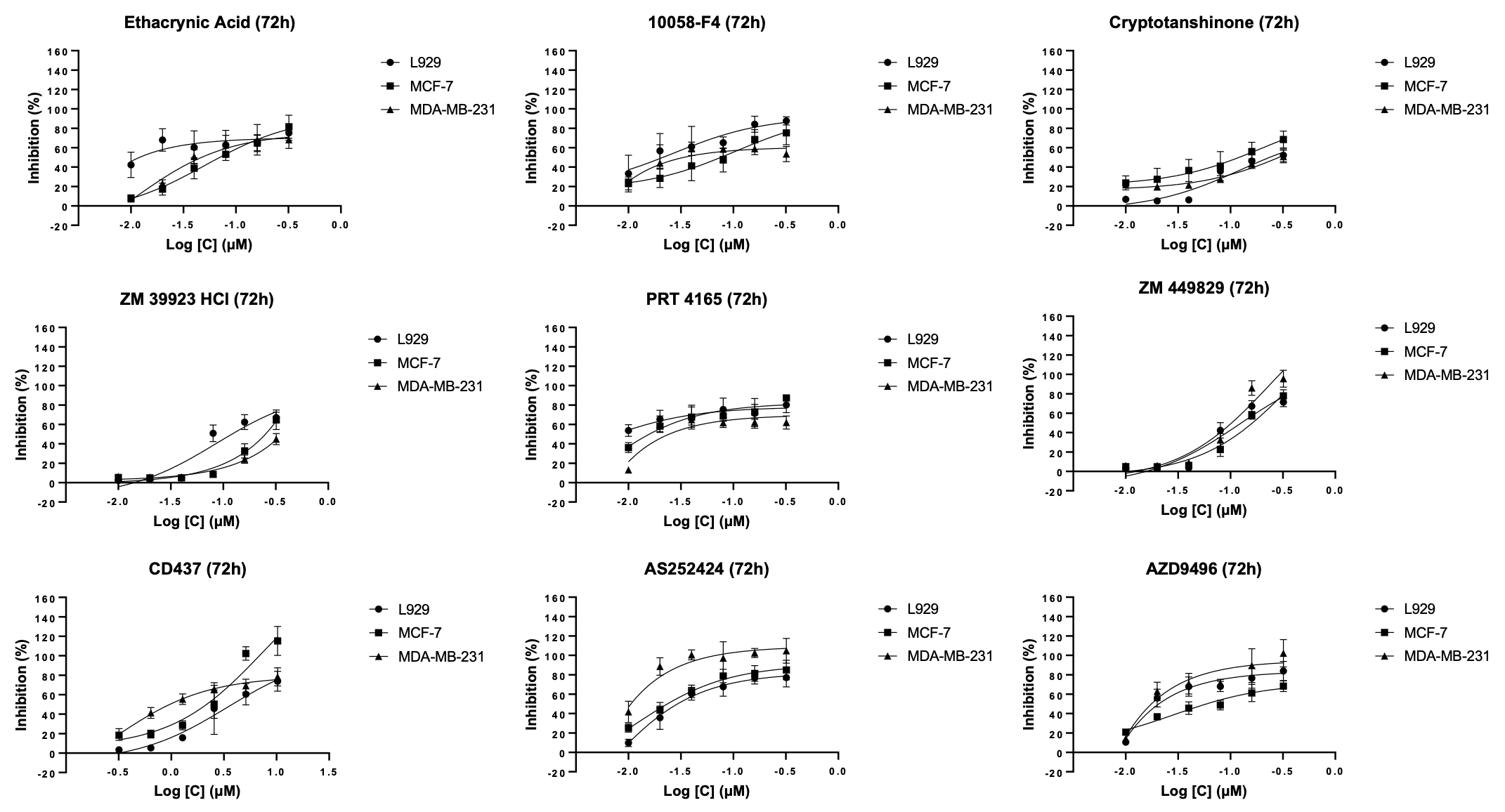
**Figure S6 –** Dose-response curves of 24 compounds in L929, MCF-7, and MDA-MB-231 cells following 72 h incubation.


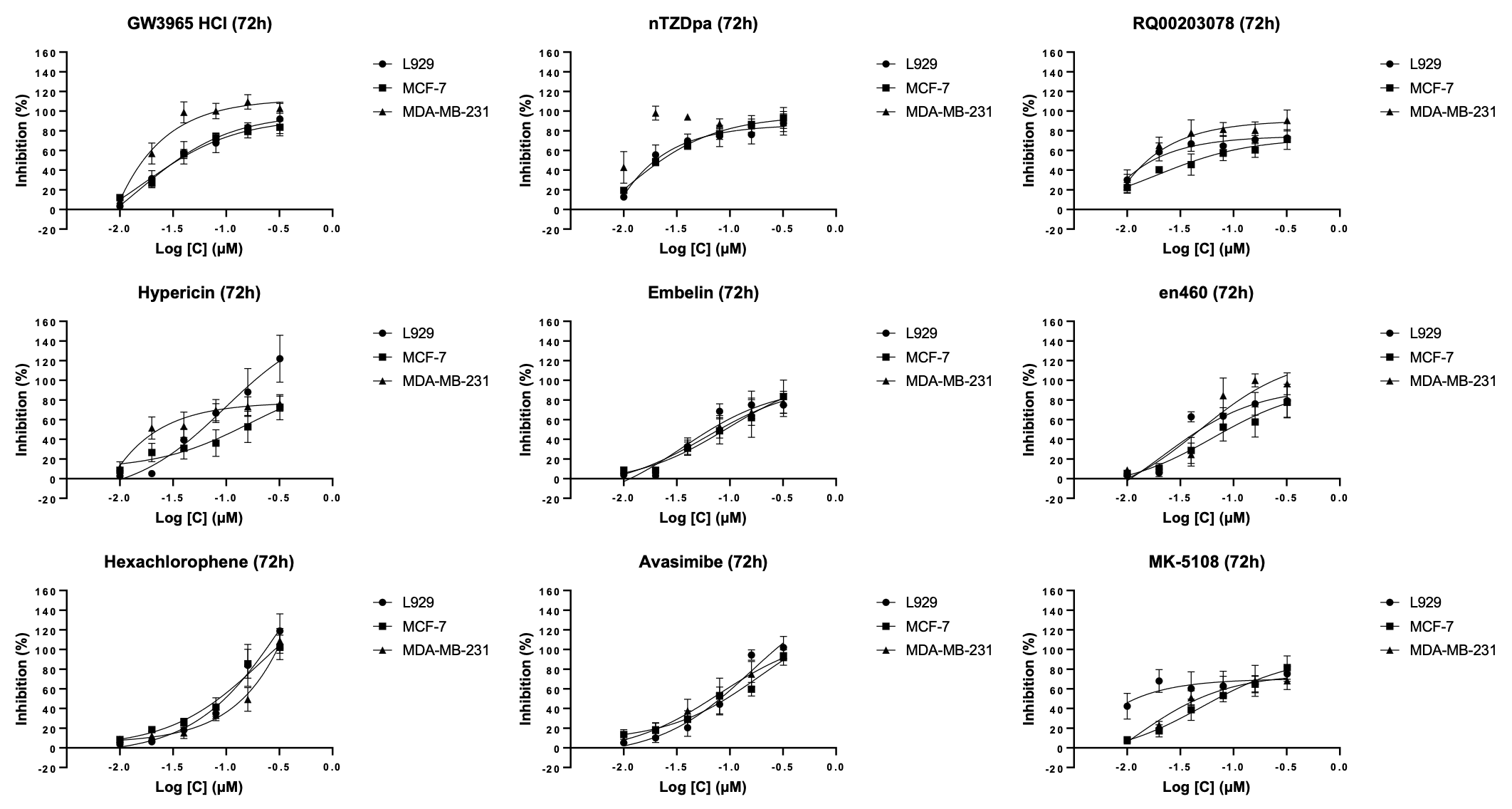


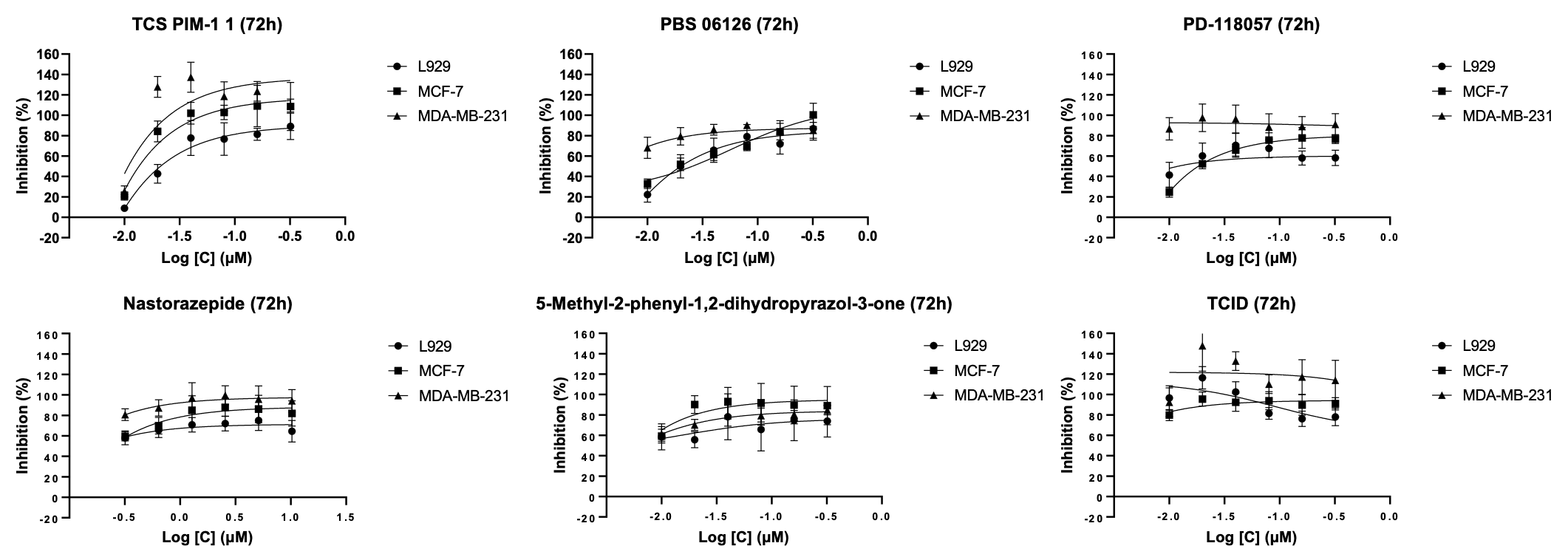


***Table S3 -*** *Synergies of drug combinations in the MCF-7 cell line.*

| **Compounds** | **Concentration IC_50_ isolated (MCF-7 cell line) (µM)** | **Combined Compounds** | | **Concentration IC_50_ combined (MCF-7 cell line) (µM)** | | **Synergism effect** |
| --- | --- | --- | --- | --- | --- | --- |
| EA | 13 |  | | | | |
| ZM 39923 | 4.3 | EA  ZM 39923 | | | 3.6  1.2 | Yes, ~ 3.6 times less |
| PRT 4165 | 59 | EA  PRT 4165 | | | 2.9  13.1 | Yes, ~ 4.5 times less |
| 10058-F4 | 14 | EA  10058-F4 | | | 4.1  4.4 | Yes, ~ 3 times less |
| Cryptotanshinone | 0.29 | EA  Cryptotanshinone | | | 3.8  0.09 | Yes, ~ 3 times less |
| ZM 39923 | 4.3 |  | | | | |
| PRT 4165 | 59 | ZM 39923  PRT 4165 | 1.3  17.8 | | | Yes, ~ 3 times less |
| 10058-F4 | 14 | ZM 39923  10058-F4 | 4.8  15.7 | | | No. |
| Cryptotanshinone | 0.29 | ZM 39923  Cryptotanshinone | 3.1  0.2 | | | No. |

***Table S4 -*** *Synergies of drug combinations in the MDA-MB-231 cell line.*

| **Compounds** | **Concentration IC_50_ isolated (MDA-MB-231 cell line) (µM)** | **Combined Compounds** | **Concentration IC_50_ combined (MDA-MB-231cell line) (µM)** | | **Synergism effect** |
| --- | --- | --- | --- | --- | --- |
| EA | 14 |  | | | |
| ZM 39923 | 2.6 | EA  ZM 39923 | | 8.2  1.5 | No. |
| PRT 4165 | 27 | EA  PRT 4165 | | 5.1  9.8 | Yes, ~ 2.7 times less |
| 10058-F4 | 15 | EA  10058-F4 | | 5.3  5.6 | Yes, ~ 2.6 times less |
| Cryptotanshinone | 2.9 | EA  Cryptotanshinone | | 3.3  0.68 | Yes, ~ 4.2 times less |
| ZM 39923 | 2.6 |  | | |  |
| PRT 4165 | 27 | ZM 39923  PRT 4165 | 2.7  28 | | No. |
| 10058-F4 | 15 | ZM 39923  10058-F4 | 5.2  29 | | No. |
| Cryptotanshinone | 2.9 | ZM 39923  Cryptotanshinone | 1.4  1.7 | | No. |
