# Supplementary figures and images for: Identification of glutathione transferase (GST P1) inhibitors via a high-throughput screening assay and implications as alternative treatment options for breast cancers
Source: PLoS One. 2025 Jul 24;20(7):e0319904. doi: 10.1371/journal.pone.0319904 (PMC12289066; doi:10.1371/journal.pone.0319904)

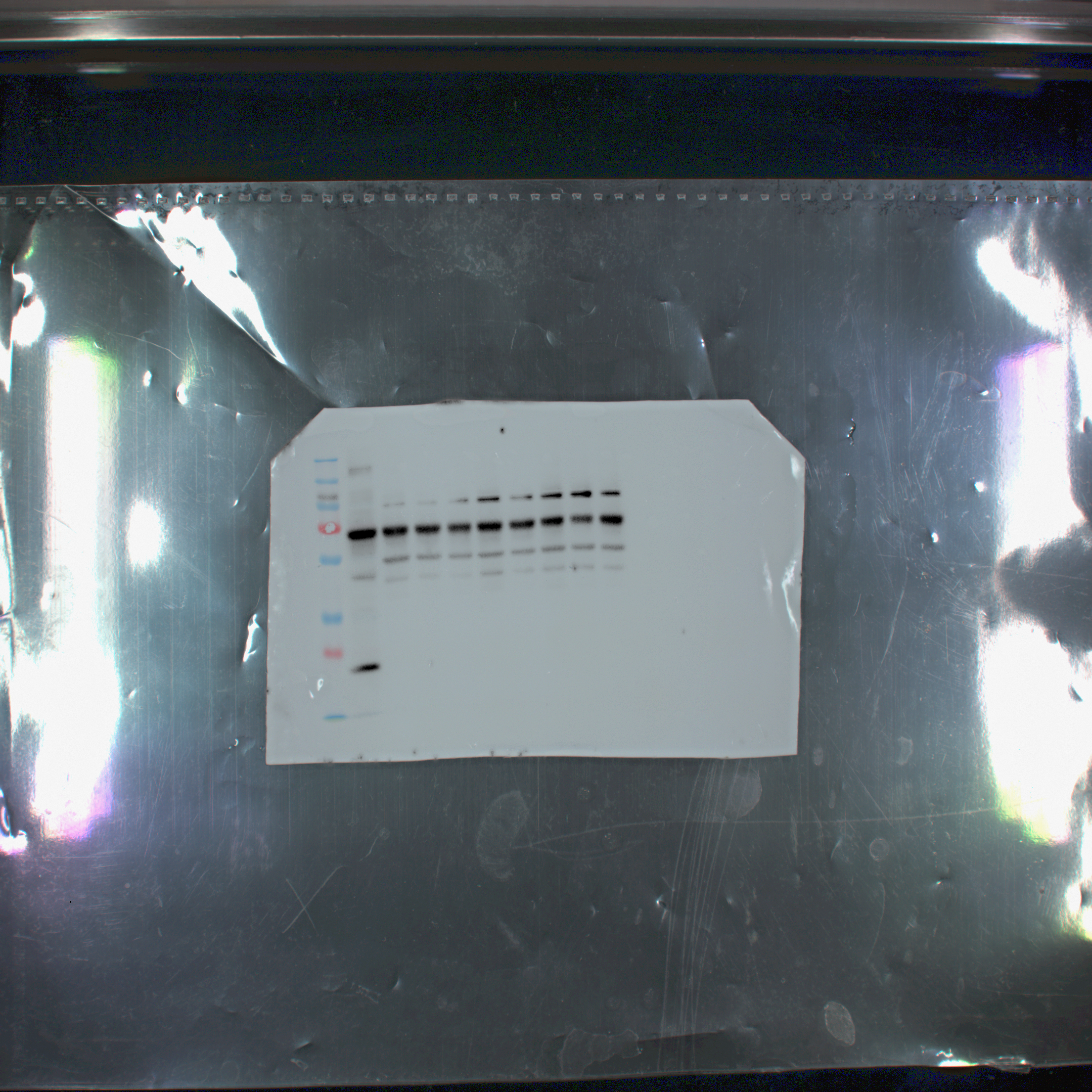

Supplement: S2 Fig — (TIF) [file pone.0319904.s002.Tif]
