# Supplementary material for: Identification of glutathione transferase (GST P1) inhibitors via a high-throughput screening assay and implications as alternative treatment options for breast cancers
Source: PLoS One. 2025 Jul 24;20(7):e0319904. doi: 10.1371/journal.pone.0319904 (PMC12289066; doi:10.1371/journal.pone.0319904)
Supplement: Attachment — Submitted filename: Response to Reviewers.docx [file pone.0319904.s005.docx]

We have modified manuscript ID PONE-D-24-21813 **“Identification of glutathione transferase (GST P1) inhibitors via a high-throughput screening assay and implications as alternative treatment options for breast cancers”**, according to all the referees’ comments. Indeed, we would like to thank the referees for their comments and suggestions that were very helpful and undoubtedly enabled us to improve the quality of the manuscript. In the revised manuscript all the changes are highlighted in yellow and they are also listed point-by-point below.

**Reviewer 1:**

**Reviewer #1: GSTs (glutathione S-transferases) are known to contribute to cancer treatment resistance by helping cancer cells detoxify anticancer drugs. Inhibiting GSTP1-1 can potentially enhance the effectiveness of anticancer therapies. This approach leverages the inhibitory properties of ethacrynic acid against GSTP1-1 to potentially improve cancer treatment outcomes. By combining ethacrynic acid with other screened compound researchers aim to create molecules that specifically target breast cancer cells.**

**Please see the following comments and revise:-**

**High-throughput GST P1-1 inhibition assay?- How it is different from regular assay? what systems were used?**

We have added the following sentence to the introduction to explain what is different about our assay compared to others in the literature:

This assay allows large numbers of compounds to be screened, using small amounts of reagents, minimizing handling steps, increasing the overall efficiency of automation.

**What is Z factor?**

We have added the following sentence to the High-throughput GST P1-1 inhibition assay section and the following reference to explain the term Z factor.

The Z-factor is a dimensionless, simple statistical characteristic for each HTS assay. This provides a valuable tool for the comparison and assessment of the quality of assays, and can be utilized in assay optimization and validation.

**Western Blot assay should be changed to Western blot Immunoassay.**

As suggested, the Western Blot assay was changed to Western blot Immunoassay.

**Figure 3 – Expression of GST P1 in MDA-MB-231 and MCF-7 cell lines. GAPDH is a housekeeping protein used as an internal reference to normalize the expression of the target protein.**

**This figure should be modified and presented well with standard protein markers**

The figure has been modified as requested. Note that the complete image was added the supporting information and a different image was added to the manuscript with the standard protein markers, see below:

**Following paper should be cited in discusion with earlier work done.**

**a) Rasha Awni Guneidy, Eman Ragab Zaki, Nevein Salah-eldin Saleh, Abeer Shokeer, Inhibition of human glutathione transferase by catechin and gossypol: comparative structural analysis by kinetic properties, molecular docking and their efficacy on the viability of human MCF-7 cells, The Journal of Biochemistry, Volume 175, Issue 1, January 2024, Pages 69–83,** [**https://doi.org/10.1093/jb/mvad070**](https://doi.org/10.1093/jb/mvad070)

The paper was cited as requested.

**Few sentences need check, repetition of words etc are found or missing words (find the attachment)**

We have carefully checked the manuscript for errors.

**Reviewer 2:**

**Reviewer #2: The manuscript entitled “Identification of glutathione transferase (GST P1) inhibitors via a high-throughput screening assay and implications as alternative treatment options for breast cancers” by Dyson et al. This study identifies 24 potent GST P1-1 inhibitors from a screen of 5,830 compounds, highlighting potential drug repurposing. Key inhibitors, including ethacrynic acid and cryptotanshinone, showed significant activity against MCF-7 and MDA-MB-231 cells. The manuscript is well-written; however there is a noticeable gap in the research. Before acceptance, the authors must address the following comments.**

**1. The manuscript is well-written; however, there are some overlapping words and punctuation errors in the text. The authors are advised to rectify these issues for enhanced clarity and coherence.**

We have rectified these issues as requested.

**2. To enhance the manuscript's comprehensiveness, include discussions on potential limitations and future research directions.**

The potential limitations and future perspectives are discussed in the Conclusions.

It is not possible to identify unexpected side effects from our studies and in the future vivo studies will be performed on promising inhibitors/combinations. Moreover, based on the extensive number of compounds screened in this study, the rational design of superior inhibitors should be feasible.

**3. Including relevant literature in the introduction will enrich the study's context and highlight its significance. Incorporating the following articles will provide valuable insights into cancer research, further supporting the study's focus.**

[**https://doi.org/10.1038/s41598-017-10864-3**](https://doi.org/10.1038/s41598-017-10864-3)

[**https://doi.org/10.1002/slct.202101853**](https://doi.org/10.1002/slct.202101853)

[**https://doi.org/10.1016/j.ygyno.2013.02.005**](https://doi.org/10.1016/j.ygyno.2013.02.005)

We are more than willing to include further relevant references, but these papers by Hussain, while very interesting, do not appear to be relevant to our study. Hence, we have not included them and would need further guidance to do so.
